# Iatrogenic anastomosis of the internal mammary artery with the great cardiac vein: The first documented case report in Palestine with a literature review

**DOI:** 10.1097/MD.0000000000043670

**Published:** 2025-08-08

**Authors:** Haya Jebreen Mohammed Warasna, Maya Sabboh, Mohammad Yaser Hasan Awad, Bashar Yaser Hasan Awad, Ahmad J. Warasna, Baha Alhadad, Qusai N. Zreqat

**Affiliations:** a Faculty of Medicine, Palestine Polytechnic University, Hebron, Palestine; b Faculty of Medicine, Tishreen University, Latakia, Syria; c Faculty of Medicine, Sumy State University, Sumy, Ukraine; d Department of Cardiology, Al-Ahli Hospital, Hebron, Palestine; e Faculty of Medicine, Al Quds University, Jerusalem, Palestine.

**Keywords:** CABG, cardiac catheterization, cardiac surgery, coil embolization, drug-eluting stent, iatrogenic

## Abstract

**Rationale:**

An unintentional anastomosis of the left internal mammary artery (LIMA) to the great cardiac vein (GCV) is a rare complication of coronary artery bypass graft surgery (CABG), resulting in a left-to-right arteriovenous shunt. It may cause angina, arrhythmias, or right-sided heart failure, with symptoms sometimes delayed for years. Management varies based on symptoms and hemodynamic impact, ranging from conservative to surgical intervention.

**Patient concerns:**

A 60-year-old male presented with recurrent exertional chest pain described as heavy, nonradiating, and relieved by rest. The pain was associated with nausea and sweating. He had a past medical history of ischemic heart disease, chronic obstructive pulmonary disease, hypertension, type 2 diabetes, hyperlipidemia, and a history of supraventricular tachycardia treated with ablation. He was a smoker and had previously undergone CABG in 2020.

**Diagnoses:**

Electrocardiography revealed ST-segment depression in leads V4–V6 and T-wave inversion in inferior leads. Troponin was negative. Coronary angiography revealed an iatrogenic LIMA–GCV anastomosis instead of the intended left anterior descending (LAD), along with severe (95%) proximal LAD stenosis and additional mid-LAD disease. Echocardiography showed preserved left ventricular function (ejection fraction 60%).

**Interventions:**

The patient underwent percutaneous transluminal coronary angioplasty with drug-eluting stents (Onyx 3.5/18 mm and Xience 2.5/18 mm) to the proximal and mid-LAD segments. No intervention was performed on the nondominant right coronary artery or the LIMA–GCV fistula during this admission.

**Outcomes:**

The patient experienced complete resolution of symptoms postintervention. The recovery was uneventful, and he was discharged on the second day of hospitalization.

**Lessons:**

Iatrogenic aortocoronary arteriovenous fistula is a rare but important complication of CABG, which can lead to significant clinical symptoms. Early diagnosis through coronary angiography is crucial for guiding appropriate management. Although percutaneous intervention with drug-eluting stents effectively resolved the patient’s angina by treating the native LAD stenosis, it is critical to recognize that this approach did not address the underlying LIMA–GCV fistula, which requires separate evaluation and management.

## 1. Introduction

Anastomosis of left internal mammary artery (LIMA) to the great cardiac vein (GCV) is an uncommon occurrence following coronary artery bypass graft surgery (CABG). A shunt from left to right is caused by an arteriovenous fistula that occurs from an unintentional anastomosis. Patients are treated conservatively,^[1]^ interventionally using coil-based embolization procedures,^[2,3]^ using detachable balloons,^[4,5]^ or by other surgical methods,^[6]^ depending on clinical symptoms and hemodynamic measurements. We describe the successful use of percutaneous transluminal coronary angioplasty with drug-eluting stents (DES) in a patient with an unintentional LIMA-GCV fistula.

## 2. Case presentation

A 60-year-old male was admitted to the hospital with recurrent chest pain, which he described as heavy, non-radiating, and worsened with exertion, but relieved by rest. This pain was accompanied by nausea and sweating. Physical examination revealed a midline chest scar from a recent CABG surgery, but no other abnormalities were found. There was no jugular venous distention or lower limb edema. His family history was positive for coronary artery disease (CAD). Electrocardiography conducted at the hospital showed ST depression in V4–V6. Subsequently, he was transferred to the critical care unit of another hospital for urgent catheterization. He had a medical history of chronic obstructive pulmonary disease, hypertension, type 2 diabetes mellitus, hyperlipidemia, and ischemic heart disease. He is a smoker (1 packet daily). His medication regimen prior to the event consisted of atorvastatin (40 mg once daily), trelegy inhaler, bisoprolol (2.5 mg once daily), dapagliflozin (10 mg once daily), and ramipril (2.5 mg once daily).

**Figure 1.**
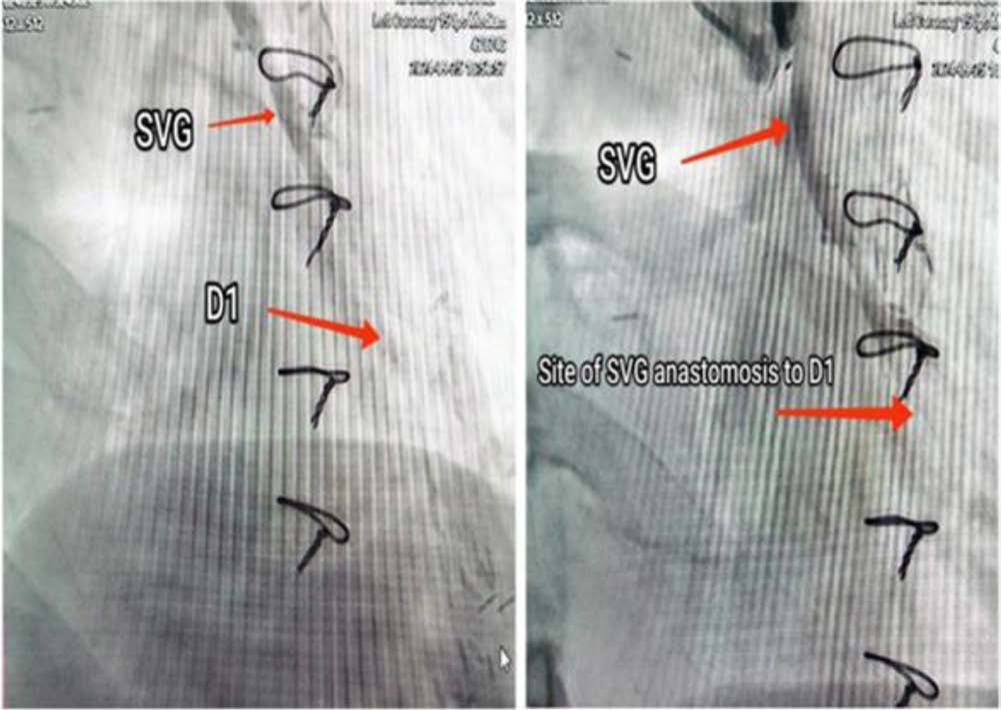
Figure shows SVG to the first diagonal branch of the left anterior descending coronary artery. D1 = first diagonal branch, SVG = saphenous vein graft.

The patient had a history of CAD for which he underwent catheterization on December 31, 2019. The patient was found to have severe proximal bifurcation disease involving the first diagonal artery branch (D1), significant ostial D1 stenosis, and mild proximal right CAD. CABG was recommended, and in January 2020, he underwent surgery with a LIMA graft to the left anterior descending artery (LAD) and a reverse saphenous vein graft to D1(Fig. 1).

In 2022, the patient was hospitalized because of recurrent palpitations, which were diagnosed as supraventricular tachycardia. The patient was referred for electrophysiological study and subsequent ablation. Electrophysiological study was conducted via the right femoral vein, and ablation was performed on the slow pathway.

Over the past 2 years, the patient has remained medically stable. Weeks before this admission (2024), he started complaining of recurrent chest pain, described as heavy. When he visited the ER, electrocardiography and troponin tests were performed. The results showed ST depression in V4–V6 with T-wave inversion in the inferior lead, and troponin was negative (<1.3 ng/L, normal <30). Subsequently, he was transferred to the critical care unit of another hospital for urgent catheterization to rule out acute coronary syndrome.

Laboratory tests revealed complete blood count, liver function tests, kidney function tests, serum electrolytes, and lipid profiles within normal limits. The patient also had normal total bilirubin and HBsAg levels. During admission, the patient received the following medications: Acetylsalicylic acid (100 mg once daily), bisoprolol (2.5 mg once daily), ramipril (2.5 mg once daily), rosuvastatin (20 mg once daily), pantoprazole (40 mg once daily), and clopidogrel (75 mg twice daily). Echocardiography showed an ejection fraction of 60% with a normal left ventricle size.

**Figure 2.**
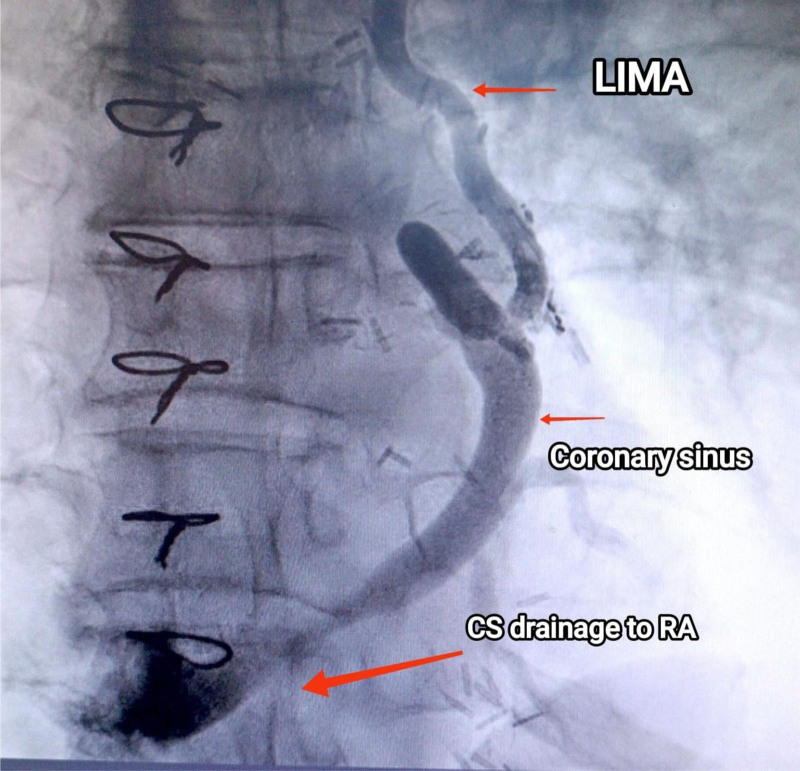
Figure shows anastomosis between left internal mammary artery and the coronary Sinus which drains into the right atrium as seen on coronary angiography. CS = coronary sinus, LIMA = left internal mammary artery, RA = right atrium.

**Figure 3.**
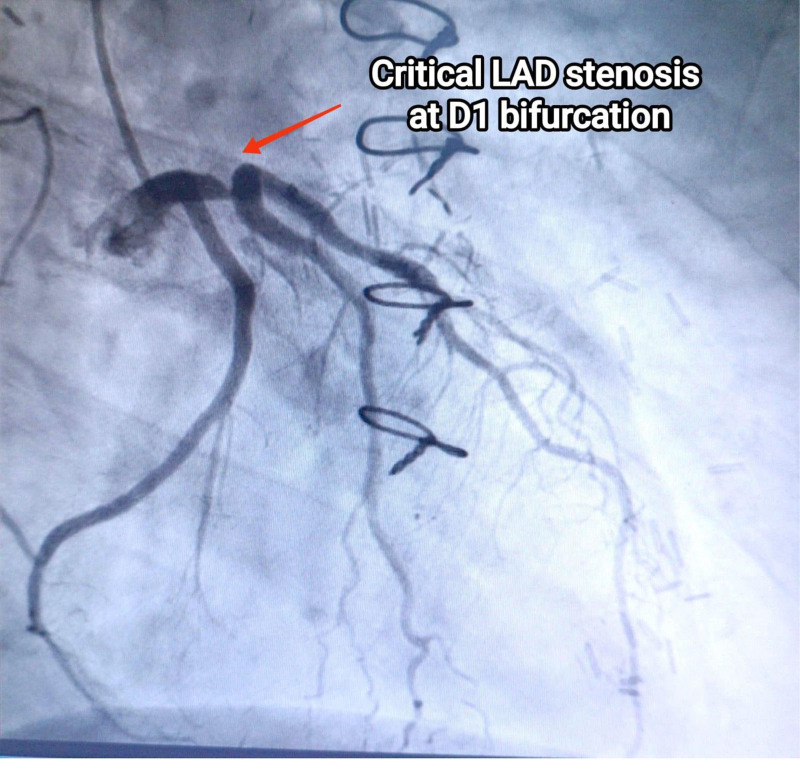
Figure shows 95% proximal left anterior descending artery stenosis at D1 bifurcation as seen on coronary angiography. D1 = first diagonal artery branch, LAD = left anterior descending.

**Figure 4.**
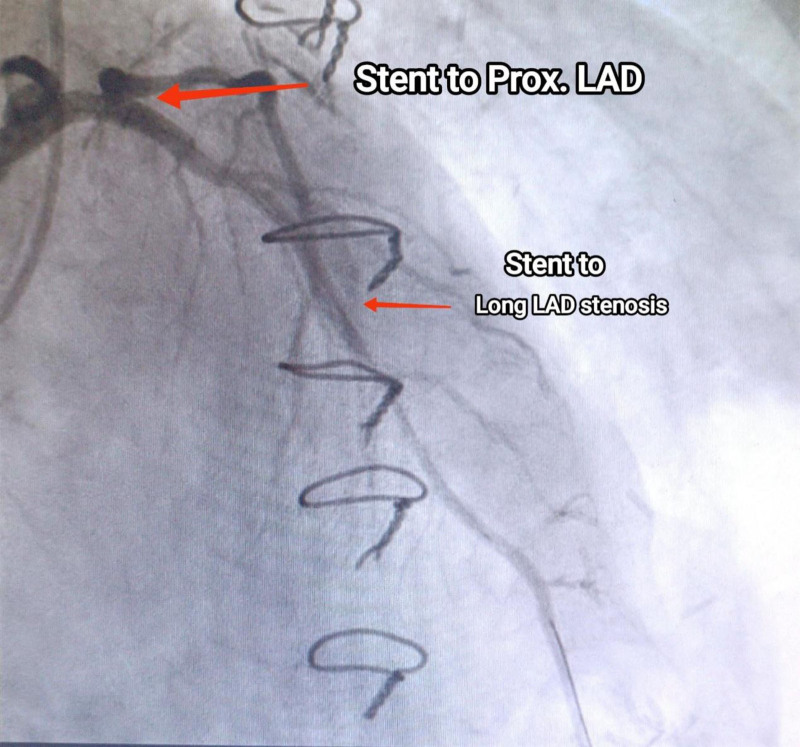
Figure shows the provisional stenting for proximal left anterior descending artery and direct drug-eluting stent the mid left anterior descending artery. LAD = left anterior descending.

**Figure 5.**
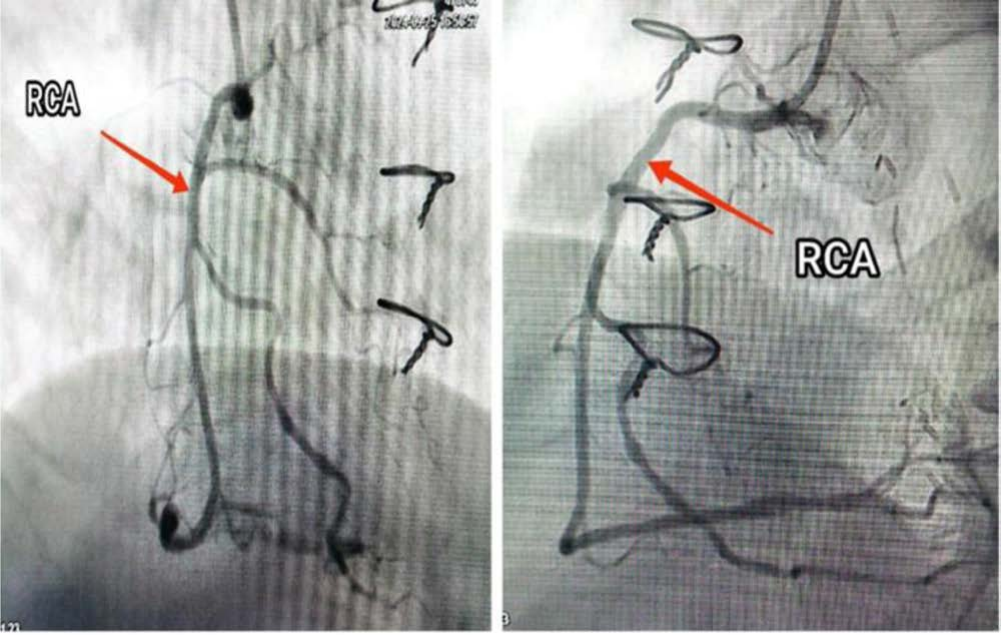
Figure shows the right coronary artery during angiography. RCA = right coronary artery.

During recent catheterization, it was unexpectedly discovered that the LIMA graft was not connected to the LAD as intended but instead to the GCV (Fig. 2). There was 95% proximal LAD stenosis at D1 bifurcation (Fig. 3), along with mild mid-atheroma at D2 bifurcation, then 70% stenosis. The intervention included percutaneous transluminal coronary angioplasty and direct DES (Onyx 3.5/18 mm) inflated to 3.9 mm in the proximal LAD (provisional stenting for proximal LAD), followed by direct DES (Xience 2.5/18 mm) inflated to 2.7 mm in the mid-LAD (Fig. 4). The right coronary artery lesion was mild (non-critical) and the vessel was non-dominant; therefore, surgical revascularization was not pursued (Fig. 5). The patient experienced an uneventful recovery and was discharged on the second day.

**Figure 6.**
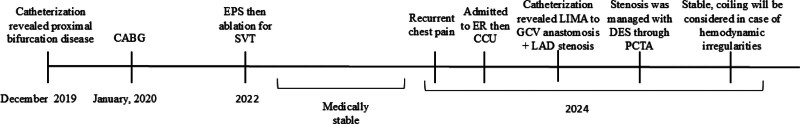
Figure shows the patient cardiac symptoms and interventions timeline. CABG= Coronary Artery Bypass Surgery, CCU = Critical Care Unit, DES = drug-eluting stent, EPS = Electrophysiological Study, LAD = left anterior descending, LIMA = left internal mammary artery, PTCA = Percutaneous Transluminal Coronary Angioplasty, SVT = Supraventricular Tachycardia.

This patient was scheduled for right- and left-sided catheterization for a better evaluation of his hemodynamics and to assess the importance of the shunt, with consideration for fistula coiling. The timeline for relevant cardiac events is depicted in Figure 6.

## 3. Discussion

An iatrogenic arteriovenous fistula is a possible complication for cardiac procedures such as CABG, percutaneous coronary intervention, and pacemaker implantation or extraction.^[7,8]^ One specific type of arteriovenous fistula is aortocoronary arteriovenous fistula (ACAVF), a rare sequela of CABG. This occurs when a graft (either the internal mammary artery or the great saphenous vein) is inadvertently anastomosed to an epicardial vein rather than its intended artery.^[9]^ In our case, the LIMA was anastomosed to the GCV. These incidents can be explained by multiple factors, such as distortion of cardiac tissues due to past infarctions or surgeries, thickening of the epicardial fat, or cases where the native artery is intramyocardial or severely atherosclerotic.^[10,11]^ in addition to the fact that CABG is usually performed under cardioplegia, which makes it difficult for the surgeon to differentiate between an intramyocardial portion of the intended artery and a nearby sclerotic vein in an area of epicardial fat.^[12]^ It is worth mentioning that LIMA can also be involved in ACAVF in rare cases where a secondary CABG is performed and a radial graft is inadvertently anastomosed to the circumflex vein instead of the LAD, thus creating a pathway between the LIMA and circumflex vein.^[13]^ There have also been cases in which LIMA was inadvertently grafted into a superficial vein on the chest^[14]^ or chest wall collateral vessels.^[15]^ Table 1 demonstrates all cases of LIMA to GCV anastomosis we found in the literature.

**Table 1:** Table demonstrates all cases in the literature describing left internal mammary artery to great cardiac vein anastomosis.

Case	Year	Patient	Symptoms	Diagnosis	Anastomosis	Management	Interval
Deligonul^[16]^	1986	66m	Asymptomatic	Coronary angiography	Anterior cardiac interventricular vein	Spontaneous occlusion after 10 yr	–
Deligonul^[16]^	1986	57m	Asymptomatic	Coronary angiography	Anterior cardiac interventricular vein	Spontaneous occlusion after 1 yr	–
Miranda^[17]^	1990	66m	Recurrence and progression of angina	Coronary angiography	Anterior cardiac interventricular vein	Balloon occlusion	2 wk
De marchena^[18]^	1990	73m	Diminishing exercise capacity and progressive dyspnea	Coronary angiography	GCV	Conservatively	2 mo
Braun^[2]^	1999	58m	Symptomatic anterior wall ischemia due to coronary steal	Coronary angiography	Internal thoracic vein	Coil embolization	6 mo
Thomas^[7]^	1999	76m	Exertional angina	Coronary angiography	Anterior cardiac interventricular vein	Embolization	3 mo
Banerjee^[19]^	2005	65f	Exertional angina and dyspnea	Coronary angiography	An epicardial vein	Redo CABG	6 yr
Khunnawat^[20]^	2006	57m	Dyspnea	Coronary angiography	GCV	Patient lost to follow-up	6 yr
Martinez^[21]^	2006	54m	Congestive heart failure and severe chest pain	Coronary angiography	GCV	Coil embolization	During the first postoperative days
Sheiban^[22]^	2006	73m	Effort angina	Coronary angiography	GCV	Percutaneous retrograde coronary sinus catheterization and covered stent deployment at the anastomotic site	2 mo
Puri^[12]^	2009	68m	New-onset angina	Coronary angiography	GCV	Successful placement of a drug-eluting stent at the native proximal lad lesion resulting in “flush” occlusion of the grafted large 1st diagonal artey branch	2 yr
Badhey^[23]^	2010	64m	Heart failure	Coronary angiography	Anterior cardiac interventricular vein	Amplatzer	3 mo
Jung^[24]^	2011	50m	Asymptomatic follow-up	Coronary CT angiography	GCV	Occluded using 3 coils	1 wk
Gardner^[25]^	2012	69m	Exertional chest pain	Coronary angiography	A cardiac vein	Stainless steel coils	3 mo
Başaran^[26]^	2012	51m	Effort dyspnea and angina	Coronary angiography	Anterior cardiac interventricular vein	An amplatzer vascular plug was deployed to the proximal segment of lima	2 mo
Lumley^[27]^	2013	70m	Progressive angina	Coronary angiography	GCV	Managed conservatively and will be followed up	1 yr
Bas^[10]^	2016	43f	Recurrent onset of angina and fatigue	Cardiac computed tomography angiography (CTA)	Proximal GCV	Coils	2 yr
Bagherli^[28]^	2017	66m	Recurrent angina and dyspnea	Coronary angiography	GCV	Percutaneous embolization using coils	Shortly after discharge
Saba^[29]^	2018	70	Dyspnea	Coronary angiography	GCV	Percutaneous coil embolization of the aberrant bypass graft	10 yr
Sreenivasan^[30]^	2018	61f	Acute onset of chest pain	Coronary angiography followed by CTA	GCV	Medical management	3 yr
Saleh^[31]^	2019	35f	Persistence of exertional angina	Coronary angiography	GCV	Not hemodynamically significant; hence no intervention was attempted	6 mo
Bravo-jaimes^[32]^	2020	71m	Progressive angina	Coronary angiography	GCV	Conservatively	3 yr
Shaikh^[33]^	2021	67m	Left-sided chest pain associated with dyspnea	Coronary angiography	GCV	Surgical ligation	2 yr
Ghumman^[34]^	2023	61m	Chest pain	Coronary angiography	Great anterior cardiac vein	Untreated with consideration of future embolization if needed	Recent CABG
Current case	2024	60m	Recurrent chest pain	Coronary angiography	GCV	Managed by placement of a DES to the proximal and mid-LAD and will be followed up for the required coiling	4 yr

CABG = coronary artery bypass graft surgery, CTA = computed tomography angiography, DES = drug-eluting stent, f = female, GCV = great cardiac vein, LAD = left anterior descending, m = male, mo = month, wk = week, yr =year.

The interval after which an anastomosis between the LIMA and GCV was discovered varied between shortly after the procedure and 12 years after it.^[28]^ With most patients reporting symptoms between 6 weeks and 4 years after surgery.^[24]^ ACAVF can be diagnosed during a regular follow-up in asymptomatic patients^[24]^ or after examining cardiac circulation in symptomatic patients.^[10]^ Diagnosis of ACAVF is usually done using coronary angiography or cardiac computed tomography angiography.^[9]^ As aforementioned, patients may be asymptomatic or symptomatic. Symptomatic patients may present with angina due to coronary steal, dyspnea, arrhythmias, syncope, fatigue or diminished exercise capacity, wide pulse pressures, new-onset murmur, or even congestive heart failure especially right-sided due to left-to-right shunt.^[9,10,23]^

Management of iatrogenic coronary arteriovenous fistula is dependent on multiple factors such as symptoms, severity of left-to-right shunt and development of volume overload in the right ventricle.^[27]^ There is no clear consensus on when a fistula should be repaired; some have suggested the presence of symptoms or the development of a significant left-to-right shunt as an indication for fistula correction but it is still a controversial topic.^[35]^ Asymptomatic cases usually have a small left-to-right shunt, which alone is not an indication for intervention, and such patients may be managed conservatively^[34]^ as they may close spontaneously, as reported by Deligonul.^[16]^ However, in some rare cases this holds a risk of the patient developing complications such as heart failure, coronary ischemia, endocarditis or fistula rupture and subsequent tamponade.^[10]^ Symptomatic cases have been managed surgically or endovascularly. Traditionally, surgery involves fistula closure and repeat bypass grafting of the initially intended vessel.^[12]^ A redo surgery however should be thoroughly calculated and preceded by several diagnostic measures to get a detailed understanding of the patient’s cardiac anatomy and vasculature.^[9]^ Another thing to keep in mind is that if a redo surgery is to be performed, the graft should be occluded before inducing cardioplegia, as keeping the graft patent will make cardiac arrest ineffective or impossible.^[9]^ Rinaldi et al also suggested open ligation of the draining vein especially if inadvertent anastomosis is discovered on the operating table.^[11]^ The surgical approach carries risks of bacterial endocarditis and worsening of pulmonary hypertension, especially in patients with unstable hemodynamics.^[23]^ A possible alternative is endovascular occlusion of the graft, which can be achieved either antegrade or retrograde. Antegrade occlusion can be performed using coils, detachable balloons, or vascular plug devices. In contrast, retrograde occlusion can be performed by deploying a covered stent in the vein at the anastomosis site.^[26]^ In cases where coils are used, it is essential to adopt techniques that mitigate coil migration, which is a lethal condition when the coronary artery is occluded. Bas et al suggested using detachable coils as well as larger-sized coils to nest smaller coils or using a balloon proximally to the coil to test its migration once the balloon is deflated.^[10]^ To avoid the risks of complete coiling, Badhey et al used an Amplatzer vascular plug, which is a less expensive alternative that does not migrate; however, using this technique may delay thrombosis of the LIMA and therefore require implantation of a second plug.^[23]^ All these considerations will be considered when the patient needs endovascular intervention.

We highlight the importance of preventing such mistakes and discovering their occurrence early. Preoperatively, angiography and thorough planning can help surgeons familiarize themselves with any peculiar anatomy that they may face. Intraoperatively, graft assessment using transit time flow measurement provides quantitative flow data and waveform analysis. Misanastomosis, such as LIMA-to-GCV, results in highly anomalous flow patterns inconsistent with arterial bypass. For instance, unexpectedly low or absent flow, atypical dampened waveforms, or an extremely high pulsatility index would strongly suggest an incorrect connection. These deviations from the expected arterial flow profiles serve as critical red flags, prompting immediate reevaluation of the anastomotic site and target vessel.^[36,37]^ These deviations from the expected arterial flow profiles serve as critical red flags, prompting immediate reevaluation of the anastomotic site and target vessel. High-resolution epicardial ultrasonography (HR-ECUS) offers real time, high-resolution imaging. While primarily used for assessing anastomotic quality and distal vessel patency, it can also reveal anatomical discrepancies indicative of misanastomosis. Visualizing a connection to a vessel lacking the characteristic features of the LAD, such as venous flow patterns (continuous, nonpulsatile) in the recipient vessel, provides unequivocal evidence of an arteriovenous connection or identifying the LAD unconnected nearby would be a clear indication of an error.^[38,39]^ Furthermore, Doppler capabilities within HR-ECUS can confirm venous flow characteristics in an unintended recipient vessel, unequivocally confirming the misanastomosis.^[40]^ In a study by Di Giammarco et al, intraoperative decisions to revise grafts were more often prompted by HR-ECUS findings than by TTFM alone. Upon reviewing 717 grafts, they discovered that the positive predictive value of TTFM increased from 10% to 100% when HR-ECUS was added to the intraoperative assessment.^[41,42]^ Therefore, the combined use of TTFM and HR-ECUS significantly increases the diagnostic accuracy of intraoperative graft verification.^[41,43]^ Budde et al demonstrated that HR-ECUS has excellent sensitivity (98%) and specificity (100%) for detecting anastomotic errors.^[44]^ In summary, although TTFM and HR-ECUS are not direct misanastomosis detectors, they provide objective, real time data that, when interpreted in context, can effectively signal an underlying error, facilitating timely corrective action. In this case, the intraoperative application of TTFM and HR-ECUS, which was not utilized during the index surgery, would likely have identified the LIMA-GCV misanastomosis through its characteristic hemodynamic and anatomical signatures, potentially preventing delayed complications. Another promising technique is intraoperative fluorescence imaging.^[45]^ Postoperatively, early computed tomography angiography can be beneficial as it detects many cardiac and noncardiac events, positively influencing clinical decisionmaking.^[46]^

## 4. Conclusion

Iatrogenic ACAVF is a rare but clinically significant complication of CABG, with presentations ranging from asymptomatic findings to severe hemodynamic compromise. This case illustrates an unusual LIMA-GCV anastomosis discovered during the evaluation of recurrent angina 4 years after CABG. Although percutaneous intervention with DES effectively addressed the patient’s symptomatic native LAD stenosis, it is critical to recognize that this approach did not treat the underlying fistula, which will require hemodynamic assessment and potential endovascular closure.

## Author contributions

**Data curation:** Baha Alhadad, Qusai N. Zreqat.

**Investigation:** Baha Alhadad, Qusai N. Zreqat.

**Project administration:** Maya Sabboh.

**Resources:** Baha Alhadad, Qusai N. Zreqat.

**Supervision:** Haya Jebreen Mohammed Warasna.

**Validation:** Haya Jebreen Mohammed Warasna, Maya Sabboh.

**Visualization:** Haya Jebreen Mohammed Warasna, Maya Sabboh, Mohammad Yaser Hasan Awad, Bashar Yaser Hasan Awad, Ahmad J. Warasna, Baha Alhadad, Qusai N. Zreqat.

**Writing – original draft:** Haya Jebreen Mohammed Warasna, Maya Sabboh, Mohammad Yaser Hasan Awad, Bashar Yaser Hasan Awad, Ahmad J. Warasna.

**Writing – review & editing:** Haya Jebreen Mohammed Warasna, Maya Sabboh, Mohammad Yaser Hasan Awad, Bashar Yaser Hasan Awad, Ahmad J. Warasna.
